# Effectiveness of the Strengthening Families Programme 10–14 in Poland for the prevention of alcohol and drug misuse: protocol for a randomized controlled trial

**DOI:** 10.1186/1471-2458-12-319

**Published:** 2012-06-20

**Authors:** Katarzyna Okulicz-Kozaryn, David R Foxcroft

**Affiliations:** 1 Institute of Psychiatry and Neurology, Sobieskiego 9, 02-957, Warsaw, Poland; 2 Oxford Brookes University, Oxford, OX3 0FL, UK

## Abstract

**Background:**

Alcohol and other drug use and misuse is a significant problem amongst Polish youth. The SFP10-14 is a family-based prevention intervention that has positive results in US trials, but questions remain about the generalizability of these results to other countries and settings.

**Methods/Design:**

A cluster randomized controlled trial in community settings across Poland. Communities will be randomized to a SFP10-14 trial arm or to a control arm. Recruitment and consent of families, and delivery of the SFP10-14, will be undertaken by community workers. The primary outcomes are alcohol and other drug use and misuse. Secondary (or intermediate) outcomes include parenting practices, parent–child relations, and child problem behaviour. Interview-based questionnaires will be administered at baseline, 12 and 24 months.

**Discussion:**

The trial will provide information about the effectiveness of the SFP10-14 in Poland.

**Trial registration:**

International Standard Randomised Controlled Trial Number: ISRCTN89673828

## Background

The European Union (EU) is the heaviest drinking region of the world, drinking 11 litres of pure alcohol per adult each year [1]. More than 1 in 4 deaths among men (aged 15–29 years) and 1 in every 10 deaths among young women in the EU is alcohol related [2]. Young people (aged 15–24 years) are responsible for a high proportion of this burden, with over 25% of youth male mortality and approximately 10% of young female mortality being due to alcohol [1]. Sparse information exists on the extent of social harm in young people, despite the fact that a third of a million (6%) 15–16 year old school students in the EU report engaging in fights, and 200,000 (4%) report unprotected sex, due to their own drinking [1].

Alcohol and other drug use increases markedly between the ages of 11 and 15 years amongst young people in Poland. Between the ages of 11 and 15 the proportion of those who have ever smoked increases from 12% to 59%. At age 11, 10% of 11-year-olds report that they have ever been drunk, and this increases to 53% amongst 15-year-olds. Moreover, 18% of Polish youth report lifetime cannabis use [3]. Early alcohol and other drug use is associated with a range of subsequent adverse health and social outcomes [4-11].

The Strengthening Families Program 10–14 (SFP10-14) is a US-developed family-based intervention for preventing alcohol and other drug use and problems amongst young people. It has been evaluated in two large-scale randomized controlled trials in Iowa, USA [12-16] and has informed the development of a family-based intervention for African American families evaluated in a large randomized controlled trial in rural Georgia, USA [17-19]. Several systematic reviews have highlighted the promising results from these trials but also note the question of whether this US-developed intervention will be applicable in other countries and settings [20-25].

### Aims of the project

The aim of the trial is to assess the effectiveness of the Polish version of the SFP10-14 (“Program Wzmacniania Rodziny”) when compared with a control group, in a large randomized controlled trial in Poland.

The objectives of this trial are:

· To examine the effectiveness of the SFP10-14 in promoting positive parenting practices in parents of 10–14 year-olds in Poland

· To examine the effectiveness of the SFP10-14 in promoting positive parent–child relations amongst families with 10–14 year-olds in Poland

· To examine the effectiveness of the SFP10-14 in reducing problem behaviour amongst 10–14 year-olds in Poland

· To examine the effectiveness of the SFP10-14 in preventing alcohol and drug use and misuse amongst 10–14 years-olds in Poland

## Methods/Design

### Design

A parallel group cluster randomized controlled trial where communities will be randomly assigned, with concealed allocation, to one of two groups, with a 1:1 allocation ratio. Communities in the intervention arm will participate in SFP10-14 group sessions; communities in the control arm of the trial will receive information leaflets for families. All families recruited into the trial will be assessed at baseline and at 12 and 24 months follow-up. Two years after baseline data collection, families from control communities will be offered the opportunity to participate in SFP10-14.

### Ethics

Research undertaken in Poland funded by the National Bureau for Drug Prevention is reviewed by independent experts, and this independent review process covers scientific and any ethical issues that are identified by the independent reviewers. Ethics Committee approval for data collection was obtained from “Komisji Bioetycznej przy Instytut Psychiatrii I Neurologii W Warszawa” (Ethics Committee of the Warsaw Institute for Psychiatry and Neurology). Each family recruited into the trial receives an information sheet describing the trial and data collection procedures before giving their written and signed consent to participate. Consent was obtained from parent(s) and, separately, from children.

### Setting and participants

Eligible participants are families with 10–14 year-old children from community settings across Poland. In all families at least one parent should agree to participate. If two children from the same family are involved in the intervention group then both parents will be asked to participate in SFP10-14 group sessions.

### Recruitment

Communities who have expressed an interest in the SFP10-14 will be approached in 2010 to participate in the trial. Information about the SFP10-14 has been disseminated throughout Poland via conferences, journal articles, information bulletins and personal contact. Within communities, families will be recruited by community workers. Family recruitment will take place through community agencies, schools and via information leaflets and personal contact.

### Randomization

Randomization occurs after communities have consented to participate in the trial. Simple randomization of community to intervention or control arm will be undertaken by the lead investigator drawing names out of a hat in a concealed allocation format.

### Intervention

The SFP10–14 is a video based programme delivered by trained facilitators that includes parents/guardians and children learning together [26-29]. The 7-week program is delivered over 7-sessions, with 4 optional booster sessions available several months later. The weekly sessions last two to three hours: in the first hour parallel groups of children and parents from up to 15 families develop their understanding and skills, led by parent and child group facilitators; in the second hour, parents and children come together in family units to practice the principles they have learned. The remaining time is spent in logistics, meals and enjoyable family activities. The programme is highly structured with detailed manuals, videos and activities whilst at the same time being highly interactive [26-29].

### Outcome measures

Alongside demographic questions (including family size and structure, parental education, work status, disposable income) we have carefully selected validated instrument measures/scales:

#### Primary outcomes

· Alcohol, cigarette and other drugs: age of first use, lifetime prevalence, 30-days (not other drugs) and 12-month prevalence

· Alcohol use without parent permission

· Drunkenness/binge drinking in past 30 days

#### Secondary outcomes

· General Child Management [30-32]

· Parent – Child Affective Quality [30,32]

· Aggressive and Hostile Behaviors in Interactions [30,32]

· Intervention-targeted Parenting Behavior scale [32,33]

· Index of Aggressive and Destructive Conduct [34,35]

· Family aggressiveness [36]

· Family togetherness [36]

· Maternal support [36]

· Parental monitoring [36]

· Time spent with Mother/Father [36]

· Family Rituals

· Family Life Questionnaire [37]

· Truancy

· School behavior grades

· Grade Point Average

· Strengths and Difficulties Questionnaire [38,39]

· Parental alcohol and cigarette use

### Data collection

Data are collected at baseline, 12 and 24 months. Interview-based questionnaires will be completed by parents and independently by children in separate rooms. In the control group one parent (or both if they express an interest) will complete the questionnaire, and if there are two children in the target age range (10–14) only the youngest will be asked to complete the questionnaire.

### Blinding

Due to the nature of the intervention blinding of participants, SFP10-14 facilitators and data collectors is not possible.

### Sample size calculation

No formal sample size calculation was undertaken but funding was requested for a sample size (N = 600 families) which was similar to that reported in other trials of the SFP10-14 [12-16]. These other trials have reported SFP10-14 effectiveness for reducing a number of risky behaviours amongst young people, including alcohol and drug use and misuse and other behavioural problems.

### Analyses

Clustering at the community and family level will be taken account of in multi-level data analysis. Statistical tests of difference in proportions or mean difference tests (or non-parametric equivalents) will be used to examine differences between intervention and control groups. Based on pilot study results [40], data will be analyzed for the whole sample and by several sub-group analyses: child age group (10–12; 13–14); family problems (violence, chronic illness, substance use problems, financial problems etc.; low vs high severity); child behaviour and emotional problems (low vs high severity). All analyses will be on an intent-to-treat basis, and both completed case analysis and multiple imputation analysis will be undertaken.

## Discussion

Social and cultural differences between the United States and European countries mean that positive results from US prevention trials may not translate to other countries. The Strengthening Families Programme 10–14 (SFP10–14) has been evaluated in several randomized controlled trials in rural Iowa in the United States and shown to be effective for delaying alcohol and drug initiation, but the extent that these results are applicable to other settings is not known.

The long-term goal of the SFP10–14 is reduced substance misuse and behaviour problems during adolescence. This is achieved through improved parental nurturing and limit setting skills, improved communication skills for both parents and young people and development of young people’s pro-social skills. These parenting skills and relationship factors are culturally universal so, in principle, the intervention should be applicable to other settings.

This cluster randomized controlled trial of the SFP10-14 is one of the first trials to test this intervention outside of the United States. As such, this is an important replication that will examine the transferability and applicability of this intervention in an international context. Evidence about the effectiveness of the SFP10-14 in a European setting might lead to better family-based prevention programmes across Europe or, conversely, will provide much needed insight into the applicability of US programmes to other countries.

## Abbreviations

SFP10-14, Strengthening Families Programme for Young People aged 10–14 and their Parents/Carers; US, United States; USA, United States of America.

## Competing interests

Foxcroft declares that Oxford Brookes University has a training consultancy service for the SFP10-14. Okulicz-Kozaryn declares that she has received payment for training SFP10-14 facilitators.

## Authors’ contributions

All authors have contributed to the development of this protocol. KO-K led on methodological development and analytical strategy and consulted DF on these aspects. KO-K and DF wrote the first draft together and all authors have approved this manuscript.

## Pre-publication history

The pre-publication history for this paper can be accessed here:

http://www.biomedcentral.com/1471-2458/12/319/prepub
